# Establishing and validating a predictive model for long-term control outcomes following orthokeratology lenses wear: a five-year cohort study

**DOI:** 10.3389/fcell.2026.1743506

**Published:** 2026-01-27

**Authors:** Zixun Wang, Xiaoling Zhang, Xiaoxue Hu, Hui Miao, Shujun Zhang, Feng Chang, Ruihua Wei, Zheng Guo

**Affiliations:** 1 Tianjin Key Laboratory of Retinal Functions and Diseases, Tianjin Branch of National Clinical Research Center for Ocular Disease, Eye Institute and School of Optometry, Tianjin Medical University Eye Hospital, Tianjin, China; 2 Handan Eye Hospital (The Third Hospital of Handan), Handan, Hebei, China; 3 Wuhan Children’s Hospital (Wuhan Maternal and Child Healthcare Hospital), Tongji Medical College, Huazhong University of Science & Technology, Wuhan, Hubei, China; 4 Department of Ophthalmology, General Hospital of the Central Theater Command of the People’s Liberation Army of China, Wuhan, Hubei, China

**Keywords:** axial length control, cohort study, logistic regression, myopia, nomogram, orthokeratology, prediction model, SHAP explainability

## Abstract

**Purpose:**

To develop and interpret a clinical prediction model for identifying children at risk of poor 5-year axial length (AL) control following orthokeratology (Ortho-K) lens wear, integrating traditional regression modeling with explainable machine learning.

**Methods:**

A total of 504 children with baseline myopia were included. The 5-year AL control outcome was defined as an AL increase of <1.0 mm (effective control, EC) or ≥1.0 mm (ineffective control, IC). Feature selection was performed using least absolute shrinkage and selection operator (LASSO), Boruta, and multivariable logistic regression. Machine learning (ML) model performance was evaluated across multiple algorithms, including logistic regression (LR), random forest (RF), support vector machine (SVM), artificial neural network (ANN), decision tree, light gradient boosting machine (lightGBM), and XGBoost. The best-performing model was visualized as a nomogram, dynamically deployed as a web-based risk calculator, and further interpreted using SHapley Additive exPlanations (SHAP) analysis.

**Results:**

Feature selection consistently identified a change in AL over the 3 years (Δ1, Δ2, Δ3), and flat E as the most stable predictors of 5-year AL control. Among all models, logistic regression achieved the best overall performance (F1 = 0.897, AUC = 0.969), while XGBoost showed the highest F1-score among ML methods. Enhancing clinical applicability and further simplifying the included parameters, the results Δ1, Δ3, and flat E still demonstrate strong predictive performance (F1 = 0.714, AUC = 0.949). The nomogram demonstrated good calibration and discrimination, with decision curve analysis confirming its clinical utility. SHAP interpretation revealed that Δ3 and Δ1 had the greatest influence on risk prediction, with a notable inflection point around 0.05 mm, beyond which the predicted risk of poor control increased sharply.

**Conclusion:**

A robust and interpretable predictive model was developed to estimate 5-year Ortho-K lens control efficacy using Δ1, Δ3, and flat E. The integrated SHAP analysis provided mechanistic insight and highlighted the clinical threshold (Δ3 = 0.05 mm) as a potential early warning indicator for suboptimal myopia control. The dynamic online nomogram enables individualized risk estimation and supports precision-guided intervention in pediatric myopia management.

## Introduction

Myopia is currently a major global public health issue (Jones et al., 2025; Chen et al., 2023; Burton et al., 2021; Holden et al., 2016). A recent study in China indicates that under current epidemiological trends, the overall prevalence rates of myopia and high myopia will reach 61.3% and 17.6%, respectively, by 2050 (Pan et al., 2025). The elongation of the axial length (AL), as a key parameter in the progression of myopia, is considered a major cause of changes in retinal structure and even irreversible damage to visual function (Tao et al., 2025; Liu S. et al., 2025; Hayashi et al., 2010). Monitoring changes in AL has become one of the key indicators for assessing the effectiveness of myopia control.

In recent years, alongside advocating lifestyle changes such as outdoor activities for children (Chen et al., 2025; Li J. et al., 2025), the range of myopia prevention and control methods has gradually expanded. These include repeated low-intensity red light therapy, defocus glasses, low-concentration atropine eye drops, multifocal soft contact lenses, and orthokeratology lens (Ortho-K lens) (Xiong et al., 2024; Liu et al., 2024; Zhang et al., 2024; Hazra et al., 2025; Queirós et al., 2025). Currently, there is a global consensus that Ortho-K lens demonstrate effective control of AL progression in myopia. Some studies have also conducted follow-up observations on AL progression after Ortho-K treatment. However, no predictions or evaluations have been made regarding the long-term effects of Ortho-K treatment (Martínez-Pérez et al., 2025; Xu et al., 2025a).

In recent years, the emergence of predictive models has enabled clinicians to assess future treatment outcomes based on patients’ existing data (Jackson et al., 2025; Huang et al., 2024; Guan et al., 2024). With the rapid advancement of machine learning (ML), quantitative performance comparisons between different models now assist clinicians in model selection decisions (Van Calster et al., 2019; Chalkou et al., 2023). While previous studies have suggested that ocular parameters may influence Ortho-K treatment efficacy, no predictive model specifically targeting the long-term efficacy of Ortho-K has been developed (Han et al., 2025).

Recent work has demonstrated that early AL changes and baseline age may provide predictive insight into Ortho-K lens treatment response; however, evidence regarding the prediction of long-term AL control based on multi-year longitudinal changes remains limited (Ruan et al., 2025). Therefore, the objective of this study is to analyze factors influencing long-term treatment outcomes using a five-year Ortho-K lens cohort from three medical centers. We will establish machine learning models for performance comparison and utilize the optimal model to develop a practical risk calculator capable of predicting long-term Ortho-K lens efficacy, thereby guiding clinical practice.

## Methods

### Study ethics and design process

**FIGURE 1:**
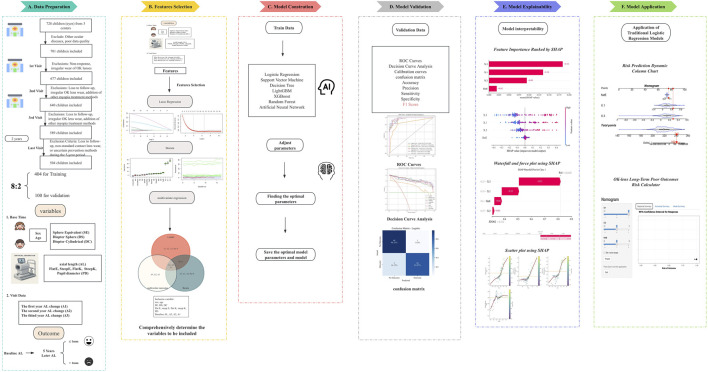
Research Design Flowchart. **(A)** Patient inclusion and exclusion process, inclusion variables, outcome variables, and dataset segmentation. **(B)** Selection of inclusion variables. **(C)** Predictive model types and development process. **(D)** Predictive model performance validation. **(E)** Explainability of machine learning contributions to model variable interpretability. **(F)** Risk stratification charts and calculator for OK-lens control efficacy at 5 years using a logistic regression predictive model based on inclusion variables.

The study was approved by the Ethics Committee of Tianjin Medical University Eye Hospital and conducted in accordance with the principles outlined in the Declaration of Helsinki (2024KY-67). Informed consent was obtained from the children and their guardians for all procedures involving ophthalmic assessments and basic Information. Figure 1 illustrates the entire workflow of this study, encompassing the following steps: inclusion and exclusion criteria, data collection, variable selection and incorporation, development of the model, performance evaluation, and interpretability of the model in the validation set.

### Data collection and preprocessing

We included basic information and baseline ocular data of pediatric patients who visited the Ophthalmology Clinic at Tianjin Medical University Hospital, Wuhan Children’s Hospital, and the Handan Eye Hospital for Ortho-K lens treatment between January 2020 and September 2025, with a minimum follow-up period of the first 3 years. The last follow-up occurred 5 years after the baseline event. Only the right eyes were analyzed to avoid interocular correlation bias (Hu et al., 2025). All children were fitted with Ortho-K (Emerald Lenses; Euclid Systems Corp, euclidsys.com, which are manufactured using BOSTON EQUALENS II (Oprifocona) material with an oxygen permeability (Dk) of 127 × 10^−11^ (cm^2^/s) (mL O_2_/mL × mmHg). The Ortho-K used in this study was a spherical four-zone lens. Each segment’s lens dimensions and curvature are individualized according to the patient’s parameters.

For baseline information on children, we included the following indicators: (1) Patient basic information: sex, age; (2) Refractive information: Cycloplegic Sphere Equivalent (SE), Cycloplegic Diopter Sphere (DS), Cycloplegic Diopter Cylindrical (DC), axial length (AL), keratometry parameters including: Steep Eccentricity (E), Flat E, Flat Keratometry (K), and Steep K, pupil distant (PD). All ocular parameters were measured three times under cycloplegia by two experienced optometrists to obtain final values. The specific cycloplegia procedure is detailed in our previously published research (Hu et al., 2025). Cycloplegic autorefraction was obtained using an autorefractor (KR-800; Topcon, Japan), and then subjective optometry was performed on the following day to get the final refraction and to calculate the SE (SER was calculated as spherical plus 1/2 columnar). AL was measured without cycloplegia using a non-contact optical biometer (Lenstar LS900; Haag-Streit AG, Haag-Streit.com). AL was measured without a washout period, consistent with established orthokeratology protocols, as corneal epithelial remodeling induced by Ortho-K is minimal in magnitude and has been shown not to affect AL assessment materially. Subsequent follow-up included only the AL measurements from the first 3 years of review, with the difference calculated from the previous year to obtain the change in AL over the 3 years (Δ1, Δ2, and Δ3). The AL examination procedure was as described above. The outcome is defined as the difference between the AL at the final follow-up and the baseline AL. Based on current consensus, we consider an average annual increase of <0.2 mm as the threshold for well-controlled myopia (Li et al., 2021; Gifford et al., 2019). Therefore, this study defines a 5-year axial length change ≤1 mm as effective control (EC) with Ortho-K lens, and a 5-year axial length change >1 mm as ineffective control (IC) with Ortho-K lens.

Exclusion criteria were as follows: (1) all subjects with missing baseline information or first 3 years AL data; (2) children with other ocular conditions such as strabismus, amblyopia, or ocular infections; (3) Children who received additional myopia control interventions alongside Ortho-K treatment (such as defocus spectacles, low-concentration atropine eye drops, repeated low-level red-light therapy and so on); (4) Children who discontinued Ortho-K lens treatment or failed to adhere to prescribed wear protocols during treatment.

### Variable selection and model development

The sample data were divided into a training set and a validation set by fivefold cross-validation sampling (training set: validation set = 8:2). The baseline variables were screened to identify potential predictors in the training set using four independent methods: multivariate regression, least absolute shrinkage and selection operator (LASSO) regression, and the Boruta algorithm. For detailed principles of each screening method, see Supplementary Material S1. We selected variables based on the intersection of key variables chosen through four methods.

In this study, 7 ML algorithms, extreme gradient boosting (XGBoost), support vector machine (SVM), Random Forest (RF), artificial neural network (ANN), decision tree, logistic regression (LR), and light gradient boosting machine (lightGBM), were used to construct the prediction model. Tenfold cross-validation was employed to ensure the model’s stability and accuracy. Grid tuning parameters were used to select the optimal settings for each algorithm. In the parameter adjustment process, the highest area under the curve (AUC) of the receiver operating characteristic (ROC) in the training data was selected as the optimal model.

### Model validation

The predictive models were trained on the designated training dataset, and the optimal model was subsequently evaluated on the validation cohort. Model performance was quantified using the AUC, accuracy, sensitivity, specificity, F1 score, Kappa, and Youden Index, with the classification outcomes visualized through a confusion matrix. Decision curve analysis (DCA) was generated to assess the clinical applicability of the models further. DCA evaluates the net clinical benefit of a predictive model across a range of threshold probabilities, thereby demonstrating its value in guiding clinical decisions (Van Calster et al., 2019; Chalkou et al., 2023). Multiple regression and ML algorithms were evaluated to capture diverse data structures, including linear effects (logistic regression), nonlinear relationships (ANN, SVM), and high-order feature interactions (tree-based ensemble models). Model selection emphasized not only discrimination but also calibration and clinical interpretability.

### Model interpretation

SHAP values were computed to quantify the contribution and relevance of each feature concerning its influence on the final classification outcome. Features with higher SHAP values were considered to exert a greater effect on the model predictions. The derived feature importance scores were presented to facilitate the interpretation of the optimal predictive model (Bhandari et al., 2022; Ukwuoma et al., 2023). Additionally, the Local Interpretable Model-Agnostic Explanations (lime) approach was applied to provide supplementary, instance-level interpretability of the model (Huang et al., 2024; Guan et al., 2024; Zhao et al., 2025; Zhou et al., 2025).

### Nomogram construction and visualization

A clinical prediction model was developed to estimate the probability of controlling myopia progression after 5 years of wearing Ortho-K lenses. Selected variables were incorporated into a logistic regression model fitted using the training dataset. A risk chart was constructed via the nomogram function to visually represent variable contributions and provide personalized risk assessment for inadequate axis length control. Predicted probabilities were converted from linear predictions via a logistic function (plogis) and calibrated across a 0.001–0.999 multi-threshold range in the validation set. Interactive dynamic nomograms were implemented using the regplot and DynNom packages. This web-based risk calculator enables clinicians to input patient-specific parameters and instantly obtain personalized predictions of long-term myopia control outcomes after 5 years of OK lens wear.

### Statistical analysis

Continuous variables were summarized as the mean ± standard deviation (SD) with ranges or the median with interquartile range (IQR), depending on the data distribution. Group comparisons were performed using Student’s t-test for normally distributed variables or the Wilcoxon rank-sum test for non-normally distributed data. Categorical variables were presented as counts and percentages, with group differences evaluated via the chi-square test or Fisher’s exact test, as appropriate. Statistical significance was defined as a two-tailed P-value <0.05. All statistical analyses were done using SPSS version 27.0 (IBM Corp.). This study used R software version 4.2.2 (R Foundation for Statistical Computing) to implement the statistical analysis. The SHAP and lime methods were completed using the “shapviz” and “lime” packages in Python version 3.10.4 (Python Software Foundation).

## Results

### Baseline characteristics

We obtained data from 728 eyes of 728 children initially recruited from three ophthalmic hospitals in China. After exclusion (Figure 1 for details), 504 eyes were ultimately included in this study. Table 1 presents the overall baseline table for this study, while Table 2 displays the univariate and multivariate regression analyses for the training set.

**TABLE 1:** Baseline information included in this study.

Varables	Level	Overall	EC	IC	p
n	​	504	348	156	​
Sex (%)	Male	230 (45.63)	156 (44.83)	74 (47.44)	0.655
​	Female	274 (54.37)	192 (55.17)	82 (52.56)	​
Age (median [IQR])	​	10.00 [9.00, 10.00]	10.00 [9.00, 11.00]	9.00 [8.00, 10.00]	<0.001
SE (median [IQR])	​	−2.75 [-4.00, −2.00]	−3.00 [-4.00, −2.00]	−2.25 [-4.00, −1.75]	0.018
DS (median [IQR])	​	−2.50 [-3.75, −1.75]	−2.75 [-3.75, −2.00]	−2.25 [-3.50, −1.50]	0.034
DC (median [IQR])	​	−0.50 [-0.75, 0.00]	−0.50 [-0.75, 0.00]	−0.50 [-0.75, 0.00]	0.44
FlatE (median [IQR])	​	0.63 [0.57, 0.69]	0.62 [0.56, 0.69]	0.64 [0.59, 0.70]	0.007
SteepE (median [IQR])	​	0.47 [0.37, 0.57]	0.46 [0.36, 0.55]	0.50 [0.39, 0.60]	0.015
FlatK (median [IQR])	​	42.85 [42.15, 43.77]	42.84 [42.20, 43.69]	42.94 [41.93, 43.94]	0.771
SteepK (mean (SD))	​	44.14 (1.37)	44.14 (1.34)	44.15 (1.43)	0.935
PD (mean (SD))	​	5.14 (0.66)	5.12 (0.62)	5.17 (0.74)	0.422
BaseAL (median [IQR])	​	24.63 [24.16, 25.18]	24.65 [24.22, 25.15]	24.56 [24.03, 25.27]	0.222
AL 1st year (median [IQR])	​	24.80 [24.39, 25.34]	24.77 [24.39, 25.26]	24.91 [24.41, 25.65]	0.087
AL 2 nd year (median [IQR])	​	25.01 [24.56, 25.54]	24.94 [24.55, 25.38]	25.22 [24.71, 25.95]	<0.001
AL 3rd year (median [IQR])	​	25.16 [24.69, 25.68]	25.01 [24.61, 25.49]	25.45 [24.93, 26.21]	<0.001
AL 5th year (median [IQR])	​	25.38 [24.89, 25.96]	25.20 [24.79, 25.69]	25.87 [25.29, 26.68]	<0.001

SE, Cycloplegic Sphere Equivalent; DS, cycloplegic diopter sphere; DC, cycloplegic diopter cylindrical; AL, axial length; steepE, steep eccentricity; Flat E, flat eccentricity; Flat K, flat keratometry; Steep K, steep keratometry; PD, pupil distant; IQR, interquartile range; EC, effective Control; IC, ineffective Control.

**TABLE 2:** Optimal parameters of each model.

Model	Optimal parameters
Decision tree	‘ccp_alpha’: 0.01, ‘max_depth’: 10, ‘max_features’: None, ‘min_samples_split’: 5
Random forest	n_estimators = 350, max_features = 2
XGBoost	‘learning_rate’: 0.1, ‘max_depth’: 5, ‘n_estimators’: 50, ‘subsample’: 1.0
LightGBM	‘colsample_bytree’: 0.8, ‘learning_rate’: 0.1, ‘n_estimators’: 100, ‘num_leaves’: 31, ‘subsample’: 0.6
SVM	‘C': 0.1, ‘degree’: 2, ‘gamma’: ‘scale’, ‘kernel’: ‘rbf'
ANN	‘activation’: ‘relu’, ‘hidden_layer_sizes’: (50, 50)

A total of 504 children who completed 5 years of Ortho-K lens wear were included in the present study (Table 1). The median baseline age of the cohort was 10.0 years IQR, 9.0–10.0), and 45.6% were boys. The median SE was −2.75 D (IQR, −4.00 to −2.00). Children in the IC group were significantly younger at baseline compared with those in the EC group (9.1 ± 1.1 vs. 9.7 ± 1.0 years, p < 0.001). No significant differences were observed between groups in terms of sex distribution, baseline SE, or DS values (all p > 0.05). In univariable logistic regression analysis, younger baseline age showed a significant association with the outcome of axial length control (OR = 0.56, 95% CI: 0.45–0.69, p < 0.001), indicating reduced odds of poor AL control relative to the reference group.

### Feature selection

**FIGURE 2:**
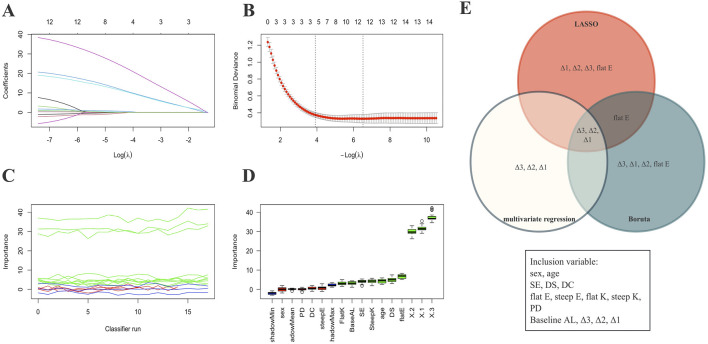
Variable selection process. **(A,B)** Visualization of feature selection using the LASSO method. **(C,D)** Visualization of feature selection using the Boruta method. **(E)** Venn diagram showing the final four variables selected for the predictive model based on three variable screening methods. X1: Δ1; X2: Δ2; X3: Δ3.

To identify the most robust predictors associated with 5-year AL control following Ortho-K lens treatment, three independent feature selection methods were applied: LASSO, Boruta, and multivariable LR (Figure 2; Supplementary Table S2). As shown in Figures 2A,B, the LASSO model minimized binomial deviance when the log(λ) value reached the optimal penalty parameter (0.0018), retaining four variables with nonzero coefficients (Δ1, Δ2, Δ3, and flat E). The Boruta algorithm further confirmed the relative importance of these features (Figures 2C,D), consistently ranking Δ3 and Δ1 as the top predictors. Multivariable regression analysis also supported their independent association with the binary outcome of AL control (p < 0.05). The intersection of the three selection strategies (Figure 2E) identified Δ1, Δ3, and flat E as the most stable and clinically meaningful predictors.

### Model development and performance comparison

**FIGURE 3:**
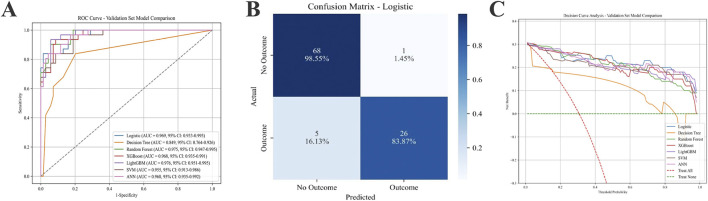
Performance comparison of seven prediction model methods on the validation set. **(A)** ROC curves for the machine learning models. **(B)** Confusion matrix for the Logistic Regression Prediction Model (validation Sets). **(C)** Seven models of the Decision Curve Analysis for the Validation Sets. XGBoost: extreme gradient boosting; SVM: support vector machine; ANN: artificial neural network; GBM: gradient boosting machine.

Seven predictive algorithms were developed to estimate the probability of effective AL control after 5 years of Ortho-K wear. Model hyperparameters were optimized via grid search and cross-validation, and the final tuned parameters for each model are summarized in Table 2. As presented in Table 3 and Figure 3, the LR model demonstrated the most balanced and robust performance, achieving an AUC (Figure 3A) of 0.969 (95% CI: 0.933–0.993), accuracy of 0.94, and the highest F1 score (0.897). This model also exhibited excellent specificity (0.986) and good sensitivity (0.839), indicating strong discriminative ability and clinical reliability (Figure 3B). The DCA curve also indicates that the RL model demonstrates superiority over other models in clinical veterinary applications (Figure 3C).

**TABLE 3:** Performance comparison of models on the validation set.

Model	AUC	95% CI lower	95% CI upper	Accuracy	Precision	Sensitivity	Specificity	F1 score	Kappa	Youden’s J
Logistic	0.969	0.933	0.993	0.940	0.963	0.839	0.986	0.897	0.855	0.824
Decision tree	0.849	0.764	0.926	0.830	0.792	0.613	0.928	0.691	0.576	0.540
Random forest	0.975	0.947	0.995	0.910	0.923	0.774	0.971	0.842	0.780	0.745
XGBoost	0.968	0.935	0.991	0.910	0.840	0.871	0.928	0.857	0.791	0.799
LightGBM	0.976	0.951	0.995	0.890	0.885	0.742	0.957	0.807	0.731	0.698
SVM	0.955	0.913	0.986	0.880	0.828	0.774	0.928	0.800	0.714	0.702
ANN	0.968	0.935	0.992	0.880	0.788	0.839	0.899	0.810	0.724	0.737

Among ML approaches, the XGBoost algorithm achieved the highest AUC (0.968, 95% CI: 0.935–0.991) and the best F1 score (0.857), followed by the RF (AUC = 0.975, F1 = 0.842) and LightGBM (AUC = 0.976, F1 = 0.807) models. Although ensemble-based ML algorithms slightly improved discrimination metrics, they exhibited lower calibration consistency and interpretability compared with logistic regression.

Considering both interpretability and clinical applicability, the LR model was ultimately selected as the final prediction model for nomogram construction and dynamic web-based risk calculator deployment. In addition, the XGBoost model—with the highest F1 score among ML methods—was subjected to SHAP analysis to quantify the contribution of key predictors (Δ1, Δ2, Δ3, and flat E) and visualize their marginal effects on the predicted risk of poor AL control.

### Interpretability analysis in the model

**FIGURE 4:**
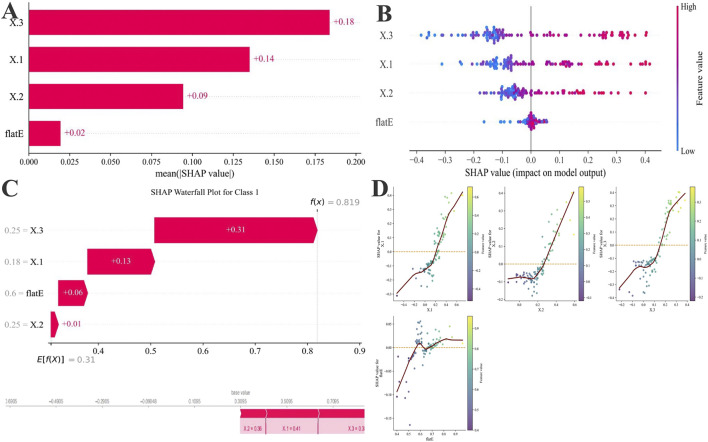
SHAP-based feature attribution for the XGBoost model. **(A)** Mean absolute SHAP values rank feature importance. **(B)** SHAP summary plot illustrating the direction and magnitude of each feature’s effect (red: high value, blue: low value). **(C)** SHAP waterfall plots for representative Class 1(PC) showing features driving risk upward (red). Below, SHAP force plots for one individual case 1, highlighting how the Δ1, Δ3, Δ2, and flat E collectively shaped prediction probabilities. **(D)** Four-variable SHAP scatter plot of total axial length change over 5 years after OK lens wear. The vertical axis represents the SHAP value (mm) for each variable’s effect on 5-year axial length change, with the red dashed line indicating SHAP = 0.

To further interpret the ML process and elucidate the influence of individual predictors, SHAP analysis was performed on the best-performing XGBoost model (Figure 4). The global feature importance ranking (Figure 4A) demonstrated that Δ3 had the greatest contribution to model output (mean SHAP = 0.18), followed by Δ1 (0.14), Δ2 (0.09), and flat E (0.02). The SHAP summary plot (Figure 4B) revealed a clear positive relationship between higher Δ3 or Δ1 values and increased probability of ineffective AL control, indicating that greater changes in these parameters were associated with suboptimal long-term outcomes. The SHAP waterfall plot (Figure 4C) illustrated how each feature cumulatively increased the prediction probability toward the poor-control class in representative samples. Notably, the SHAP dependence plots (Figure 4D) demonstrated distinct nonlinear relationships between Δ2, Δ3, and the model output. An apparent inflection point was observed at approximately 0.05 mm, beyond which the predicted risk of poor axial elongation control increased sharply. Δ3 exhibited a more pronounced inflection and stronger impact on model output compared with Δ2.

### Nomogram construction and validation

**FIGURE 5:**
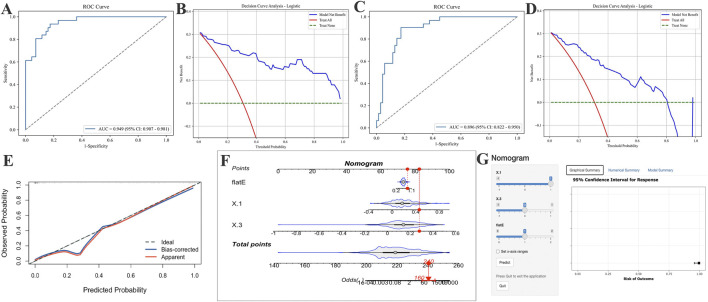
Application of AUC curves and DCA curves after incorporating different parameters into logistic regression models and establishing optimal logistic regression models for risk prediction. **(A,B)** Include only the AUC and DCA curves of the logistic regression model incorporating the change values from Δ1, Δ3 and the flat E. **(C,D)** Include only the AUC and DCA curves of the logistic regression model incorporating Δ1, Δ2, and flat E. **(E)** The final logistic regression model incorporating Δ1, Δ3, and flat E produced the calibration curve on the validation set. **(F,G)** Risk prediction nomogram **(F)** and risk calculator **(G)** for AL control efficacy after 5 years of OK-Lens wear in optimal model construction.

Considering the practicality and clinical applicability of the model, we employed LR as the optimal model and retrained it using Δ1, Δ3, and flat E, as well as Δ1, Δ2, and flat E, respectively, before evaluating the models on an independent validation set (Figure 5). Results indicate that the Δ1, Δ3, and flat E predictor model (Figures 5A,B) (Accuracy: 0.840, Precision: 0.800, Sensitivity: 0.645, F1 Score: 0.714, Specificity: 0.928, Kappa: 0.605, Youden’s J: 0.573) outperformed models built with Δ1, Δ2, and flat E as predictors (Figures 5C,D) (Accuracy: 0.800, Precision: 0.704, Sensitivity: 0.613, F1 Score: 0.655, Specificity: 0.884, Kappa: 0.515, Youden’s J: 0.497).

Based on the optimal LR model, a clinical nomogram was constructed incorporating three independent predictors (Δ1, Δ3, and flat E) to estimate the probability of achieving effective AL control after 5 years of Ortho-K lens wear. Each predictor was assigned a weighted score proportional to its regression coefficient, and the total score corresponded to the individual probability of poor AL control. Calibration and discrimination assessments confirmed that the nomogram exhibited excellent agreement between predicted and observed outcomes. The calibration curve demonstrated a close alignment with the 45° reference line, and the Hosmer–Lemeshow goodness-of-fit test indicated no significant deviation (p = 0.84, p > 0.05) (Figure 5E). To enhance clinical usability, a dynamic, web-based risk calculator was developed and deployed, allowing clinicians and patients to intuitively estimate individual 5-year AL control probabilities by entering the three model variables. This interactive tool enables personalized visual feedback and facilitates precision guidance for myopia management during Ortho-K lens treatment (Figures 5F,G).

## Discussion

This study incorporated the first 3 years’ AL changes and baseline flat E to develop a clinical prediction model for assessing myopia progression control after 5 years of Ortho-K lens wear. The results confirm that early AL changes, particularly Δ1 and Δ3, serve as robust prognostic indicators of long-term Ortho-K treatment efficacy. The model demonstrated that Δ1 was a key influencing factor, while stabilization of AL in the third year further signaled effective long-term control. A response magnitude of approximately 0.05 mm emerged as a clinically meaningful threshold, beyond which long-term control efficacy declined. To enhance clinical applicability, the final model retained only Δ1 and Δ3 along with baseline flat E, maintaining strong predictive performance and clinical value. These findings emphasize that regular AL monitoring during Ortho-K treatment provides stronger prognostic information than baseline measurements alone.

The univariate analysis revealed significant effects for Δ1, Δ2, Δ3, flat E, and age. However, when the AL change was included in the multivariate analysis, flat E and age lost statistical significance. This phenomenon may be explained by age-related physiological variations in axial length growth, whereby AL elongation gradually decelerates with increasing age in both pre-myopic and myopic children (Liu W. C. et al., 2025; Wong et al., 2025). The dynamic AL change variables (Δ1, Δ2, and Δ3) likely embed these age-related growth effects. Although the strong influence of Δ1–Δ3 diluted the independent contribution of flat E in multivariate regression, both LASSO analysis and the Boruta algorithm consistently identified flat E as a relevant predictor. Accordingly, flat E was retained in the final model. Prior work by Yang HW et al. has demonstrated that flat eccentricity provides meaningful predictive and clinical insights for orthokeratology lens fitting (Yang et al., 2025), lending theoretical support to its inclusion. Although cycloplegic refractive parameters were collected at baseline, they were not retained in the final model because refractive status during orthokeratology treatment is directly modified by corneal reshaping and may be partially compensated by age-related immortalization. AL, in contrast, reflects true structural eye growth and is therefore considered a more robust and treatment-independent outcome measure for long-term myopia control. Our findings are consistent with a recent study by Ruan et al., which reported that early AL changes and age are informative predictors of Ortho-K lens efficacy (Ruan et al., 2025). Extending this work, the present study demonstrates that dynamic AL changes across the first 3 years, particularly third-year stabilization, provide additional prognostic value for predicting five-year AL control.

In the comparative evaluation of predictive models, logistic regression demonstrated the most balanced and robust performance on the validation set. This advantage may be attributed to the longitudinal nature of the predictors and the relatively low dimensionality of the dataset. Similar methodological choices have been reported previously: Xiao Z et al. employed logistic regression to investigate factors influencing myopia severity and AL (Xiao et al., 2025), while Tao Z et al. developed a personalized myopia progression prediction model based on AL progression and baseline AL-to-corneal curvature ratio (AL/CR) (Tao et al.). Moreover, Lai H et al. showed that traditional statistical models can outperform complex machine learning algorithms in predictive accuracy when handling structured clinical data (Lai et al., 2024). Together, these findings suggest that, in certain clinical contexts, LR offers practical advantages over more complex ML approaches, particularly with respect to stability, calibration, and interpretability.

Beyond model performance, SHAP-based explainability analysis provided important insights into the contribution and nonlinear behavior of individual predictors within the XGBoost model. Among all features, Δ3 exhibited the highest SHAP importance, followed by Δ1 and Δ2, indicating that annual axial length changes, especially during the third year of Ortho-K lens wear, play a decisive role in determining long-term treatment outcomes. Supporting this observation, a five-year longitudinal study by the Jinhua Bao team reported that the reduction in myopia progression and axial elongation observed over 5 years with HAL spectacles was comparable to that achieved within the first 3 years (Li et al., 2025a), which may help explain the pronounced prognostic value of Δ3 in our cohort.

At present, evidence suggests that baseline ocular parameters may be associated with long-term Ortho-K efficacy (Ai et al., 2025), and genetic factors are known to play an important role in the onset and progression of myopia (Li et al., 2025b). Nevertheless, the effectiveness of orthokeratology varies across populations, and the mechanisms underlying this heterogeneity remain incompletely understood. A key finding of the present study is that, regardless of baseline parameter ranges, predictive performance was substantially enhanced by the inclusion of dynamic AL monitoring variables. This highlights the pivotal role of longitudinal biometric assessment and underscores the importance of sustained follow-up in pediatric Ortho-K wearers. Notably, treatment response during prolonged wear strongly predicted long-term outcomes, further reinforcing the clinical value of continuous axial length monitoring in this population.

By integrating SHAP interpretability with ML prediction, our study bridges the gap between statistical accuracy and clinical understanding, allowing the model to provide both individualized risk estimation and biologically interpretable decision cues. This explainable framework enhances trust in model outputs and offers a practical tool for precision-guided myopia management. These findings echo longitudinal clinical observations that the myopia-control effect of Ortho-K tends to plateau after 2–3 years of continuous wear. Early recognition of excessive Δ3 changes (>0.05 mm) may therefore help clinicians identify children at higher risk of accelerated axial growth and prompt timely intervention, such as refitting lenses, reinforcing outdoor activity, or combining with low-dose atropine therapy.

To further enhance clinical applicability while accounting for follow-up duration and real-world constraints, we avoided blindly pursuing maximal AUC values. Instead, we constructed simplified logistic regression models incorporating Δ1, Δ3, and flat E, as well as Δ1, Δ2, and flat E. Although the Δ1, Δ3, and flat E model performed slightly worse than the full Δ1, Δ2, Δ3, and flat E model, its validation performance remained robust. Consistent with this approach, Yang X et al. reported minimal incremental benefit when incorporating prior two-year AL changes into predictive models (Xu et al., 2025b). Based on these findings, we optimized the model of Δ1, Δ3, and flat E and developed a dynamic nomogram and risk calculator. This optimization significantly enhanced the model’s practical clinical value and demonstrated greater translational significance. Physiological AL growth was accounted for through dynamic AL change variables, which integrate age-related growth and treatment response, enabling differentiation between stable and progressive myopia trajectories.

Several limitations should be acknowledged. First, due to the extended follow-up period and limited sample size, validation was performed using a multicenter internal cohort rather than an independent external dataset. Future studies should expand sample size and incorporate external validation. Second, the current model includes a limited number of predictors; incorporation of additional behavioral, environmental, or genetic factors may further improve performance. Additionally, dynamic algorithms remain the primary development direction for myopia prediction. Future work will focus on collecting additional longitudinal data to enable outcome prediction based on AL growth at any given follow-up year. Importantly, the present model is particularly suited to Ortho-K, where refractive outcomes are masked by corneal reshaping, and AL remains the most reliable indicator of treatment efficacy. Finally, longitudinal corneal topography or epithelial thickness data were not uniformly available across the extended follow-up period, a common limitation of long-term retrospective orthokeratology cohorts. Therefore, the potential influence of corneal remodeling could not be directly quantified (Alharbi and Swarbrick, 2003).

## Conclusion

We established and validated a clinically interpretable prediction model to assess 5-year AL control outcomes in children wearing orthokeratology lenses. By combining Δ1, Δ3, and flat E as core predictors, the LR-based nomogram achieved high accuracy and good calibration, and its web-based implementation facilitates individualized clinical application. Among all evaluated algorithms, logistic regression provided the most stable overall performance, while XGBoost achieved the highest F1 score and offered mechanistic transparency through SHAP analysis. The SHAP findings revealed nonlinear feature effects and identified a clinically meaningful inflection point at approximately 0.05 mm, particularly for Δ3, underscoring the third year as a critical period for maintaining axial growth control. Therefore, compared to baseline measurements, dynamic axial length monitoring is crucial for evaluating the long-term therapeutic efficacy of Ortho-K lenses. These results not only advance the understanding of Ortho-K lens treatment dynamics but also provide a practical, explainable, and user-friendly decision-support tool for precision myopia management in children.

## Data Availability

The raw data supporting the conclusions of this article will be made available by the authors, without undue reservation.
