# Supplementary material for: Establishing and validating a predictive model for long-term control outcomes following orthokeratology lenses wear: a five-year cohort study
Source: Front Cell Dev Biol. 2026 Jan 27;14:1743506. doi: 10.3389/fcell.2026.1743506 (PMC12886394; doi:10.3389/fcell.2026.1743506)
Supplement: Supplementary file 1 [file Table1.docx]

## Table S1. Comparison of Feature Selection Methods

| Method | Principle / Algorithm | Advantages | Limitations |
| --- | --- | --- | --- |
| Univariate Selection (“Single-variable first”) | Each variable is tested individually (e.g., t-test, ANOVA, or univariate regression) to assess its association with the outcome. | Simple, interpretable, and computationally efficient. | Ignores interactions and correlations between variables. |
| Multivariate Selection | All significant variables from univariate analysis are entered into a multivariate model; non-significant predictors are removed stepwise. | Accounts for confounding and collinearity. | May still risk overfitting if too many predictors remain. |
| LASSO Regression (L1 regularization, 10-fold CV) | Applies L1 penalty to shrink coefficients; some become zero, thus performing variable selection. Optimal λ determined by 10-fold cross-validation. | Performs simultaneous variable selection and regularization; reduces overfitting. | Sensitive to highly correlated predictors. |
| Boruta Algorithm | Random forest–based wrapper method comparing real feature importance with “shadow” (permuted) features to select relevant ones. | Captures nonlinear relationships; robust to noise and interactions. | Computationally intensive; may retain redundant features. |
| Recursive Feature Elimination (RFE) | Iteratively removes least important features based on model weights or importance scores until optimal subset is achieved. | Model-driven, flexible, and effective in reducing dimensionality. | Time-consuming; performance depends on model choice. |

The Lasso regression model selects features with non-zero coefficients as potential predictors, effectively reducing multicollinearity and preventing over-fitting. We employed Lasso regression with 10-fold cross-validation to analyze the initial high-dimensional dataset and identify relevant variables. The Boruta algorithm is a feature selection technique based on variable importance. Specifically, it determines the most significant features by comparing the Z-values of candidate features with those of "shadow features." The Z-value of each fundamental feature is derived from a random forest classifier in each iteration. In contrast, the Z-value of each shadow feature is generated by randomly shuffling the real features. This method iteratively eliminates less important features than the randomly shuffled features. Consequently, only features with Z-values exceeding the maximum Z-value of shadow features in multiple internal bootstraps are retained. Regarding recursion elimination, predictor selection for model construction followed a two-stage protocol: importance ranking and sequential forward inclusion. First, a preliminary Random Forest (RF) classifier was used to quantify the contribution of each variable, retaining all features. Next, hierarchical clustering was applied to eliminate multicollinear redundancies. The surviving candidates were re-ordered, after which an iterative loop was performed across several ML classifiers, adding one predictor at a time until the final set was assembled.

## Table S2. Single-factor and Multi-factor Regression Analysis of the Training Set

| Varables |  | GC (N=279) | PC (N=125) | OR (univariable) | OR (multivariable) |
| --- | --- | --- | --- | --- | --- |
| sex | male | 120 (43%) | 60 (48%) |  |  |
|  | female | 159 (57%) | 65 (52%) | 0.82 (0.54-1.25, p=.351) |  |
| age | Mean ± SD | 9.7 ± 1.0 | 9.1 ± 1.1 | 0.56 (0.45-0.69, p<.001) | 0.82 (0.53-1.26, p=.369) |
| SE | Mean ± SD | -3.1 ± 1.3 | -2.9 ± 1.6 | 1.14 (0.97-1.33, p=.112) |  |
| DS | Mean ± SD | -2.9 ± 1.2 | -2.7 ± 1.5 | 1.13 (0.95-1.33, p=.157) |  |
| DC | Mean ± SD | -0.5 ± 0.6 | -0.6 ± 0.7 | 0.89 (0.63-1.26, p=.520) |  |
| flatE | Mean ± SD | 0.6 ± 0.1 | 0.7 ± 0.1 | 43.42 (4.31-437.90, p=.001) | 38.23 (0.24-6041.86, p=.158) |
| steepE | Mean ± SD | 0.5 ± 0.2 | 0.5 ± 0.2 | 3.14 (0.98-10.04, p=.053) |  |
| FlatK | Mean ± SD | 42.9 ± 1.1 | 43.0 ± 1.2 | 1.06 (0.88-1.28, p=.523) |  |
| SteepK | Mean ± SD | 44.1 ± 1.3 | 44.2 ± 1.4 | 1.06 (0.90-1.24, p=.467) |  |
| PD | Mean ± SD | 5.1 ± 0.6 | 5.2 ± 0.7 | 1.15 (0.82-1.59, p=.419) |  |
| BaseAL | Mean ± SD | 24.7 ± 0.7 | 24.6 ± 0.9 | 0.83 (0.63-1.09, p=.176) |  |
| Δ1 | Mean ± SD | 0.1 ± 0.2 | 0.4 ± 0.2 | 3881.06 (653.70-23042.18, p<.001) | 2282644.12 (22213.11-234567116.24, p<.001) |
| Δ2 | Mean ± SD | 0.2 ± 0.1 | 0.3 ± 0.1 | 956725.34 (55123.82-16604859.63, p<.001) | 221209019.02 (398686.50-122736611851.06, p<.001) |
| Δ3 | Mean ± SD | 0.1 ± 0.1 | 0.3 ± 0.1 | 61097810.69 (1686982.31-2212792892.98, p<.001) | 293559069558.46 (135323686.04-636820721041589.12, p<.001) |

SE: Cycloplegic Sphere Equivalent ; DS: Cycloplegic Diopter Sphere; DC: Cycloplegic Diopter Cylindrical; AL: axial length; steepE: Steep Eccentricity, Flat E: Flat Eccentricity, Flat K: Flat Keratometry; Steep K: Steep Keratometry; PD: pupil distant; IQR: interquartile range; GC: Good Control; PC: Poor Control
